# Microsurgical versus endoscopic surgery for non-functioning pituitary adenomas: a retrospective study

**DOI:** 10.3325/cmj.2020.61.410

**Published:** 2020-10

**Authors:** Tomáš Česák, Pavel Póczoš, Jaroslav Adamkov, Petr Čelakovský, Filip Gabalec, Jiří Soukup, Radka Dvořáková, Petr Krůpa

**Affiliations:** 1 Department of Neurosurgery, University Hospital Hradec Kralove, Hradec Kralove, Czech Republic; 2 Department of Otorhinolaryngology and Head and Neck Surgery, University Hospital Hradec Kralove, Hradec Kralove, Czech Republic; 3 4th Department of Internal Medicine, University Hospital Hradec Kralove, Hradec Kralove, Czech Republic; 4 The Fingerland Department of Pathology, University Hospital Hradec Kralove, Hradec Kralove, Czech Republic; 5 Department of Radiology, University Hospital Hradec Kralove, Hradec Kralove, Czech Republic

## Abstract

**Aim:**

To compare microsurgical technique (mTSS) and endoscopic technique (eTSS) in the treatment of non-functioning pituitary adenomas (NFPAs).

**Methods:**

We retrospectively evaluated the charts of 50 patients who underwent either mTSS or eTSS for NFPA in the Department of Neurosurgery, University Hospital Hradec Kralove from 2013 to 2019. We enrolled all patients who were not treated by postoperative adjuvant radiotherapy and who underwent at least two regular postoperative magnetic resonance imaging (MRI) tests. We compared the groups in terms of the extent of resection, surgery duration, blood loss, complication rate, overall clinical effect on the endocrinological and ophthalmological deficit, and postoperative growth pattern of the residual tumor mass.

**Results:**

The mTSS group had significantly shorter surgical time (75 min vs 127 min, *P* < 0.001) and lower perioperative blood loss (156 mL vs 256 mL, *P* = 0.027). The groups did not significantly differ in the extent of resection, overall clinical or hormonal effect, and the complication rate. The extent of resection did not correlate with tumor consistency, while the tumor growth rate did not correlate with age or Ki-67 expression.

**Conclusions:**

There was no major difference between the approaches in surgery radicality or safeness. However, eTSS remains the method of choice due to its potentially higher postoperative preservation of hormonal functions.

Pituitary adenomas are benign tumors representing around 10%-15% of all intracranial tumors, with a peak incidence in the third or fourth decade of life and no sex predominance. They are the third most common intracranial neoplasm in the elderly (1-3). Non-functioning pituitary adenomas (NFPAs) comprise 25%-40% of all pituitary neoplasms and are characterized by the absence of clinically measured hypersecretion of hypophyseal hormones (4-6). The annual incidence of NFPAs is 0.65-2.34/100 000 patients, with a prevalence of 7-41/100 000 cases (7-9).

Treatment options for patients with NFPAs include careful surveillance with watchful waiting, radiotherapy, or surgery. Surgical treatment, as a first-line therapy, is performed in patients with larger tumors and signs and symptoms arising from compression. The most frequently used surgical approach is transsphenoidal surgery (TSS). Microscopic transnasal surgical technique (mTSS) has for many years been the gold standard in the treatment of pathologies in the sellar region (10-13). Endoscopic transnasal approach (eTSS) was introduced in the early 1990s and has quickly developed in the last decade, mainly due to improved quality of visualization (14-21). Despite a lack of high-quality comparison of the efficacies of the two approaches, endoscopic surgery was adopted in a vast majority of neurosurgical centers, including ours. However, the overall advantages and disadvantages of both techniques are still a matter of debate, as most of studies yielded controversial conclusions resulting from various research methods.

The aim of our study was to compare the two surgical methods for the treatment of NFPAs. We evaluated the extent of resection, safety, surgery duration, blood loss, and the overall clinical effect on endocrinological and ophthalmological deficit. We also analyzed how surgical radicality depended on the consistency/invasiveness of the tumor mass and assessed the correlation of the postoperative growth pattern of the residual tumor mass with age and Ki-67 index.

## Patients and methods

We retrospectively reviewed the charts of 50 patients who had undergone either mTSS or eTSS for NFPA (Hardy 0-IV, Knosp 0-3) without adjuvant radiotherapy in the Department of Neurosurgery, University Hospital Hradec Kralove between January 2013 and January 2019. Each group comprised of 25 patients. Endoscopic technique was chosen for patients with more extrasellar tumor propagation and was preferred mainly in the past three years. All the operations were performed by the first author, who is versed in both techniques. mTSS was performed by two neurosurgeons, and eTSS by two neurosurgeons assisted by an otorhinolaryngology surgeon, who performed the endoscopic fenestration of the sphenoidal sinus. All patients were followed-up for a minimum of 12 months. The follow up visits were scheduled at 3 and 12 months post-operatively and involved magnetic resonance imaging (MRI) and endocrinological and ophthalmological examination. According to preoperative MRI, adenomas were classified according to standard classification systems (11,22). During the operation, tumor consistency (soft, stiff, mixed), surgical time, and blood loss were assessed. The extent of the resection was evaluated by postoperative coronal MRI T1 images using graphics software (Brainlab, Munich, Germany) and graded as gross total resection (GTR, no macroscopic residuum), near-total resection (NTR, ≥95% of the preoperatively visualized mass), and sub-total resection (STR, <95% of the preoperatively visualized mass). During the follow-up, postoperative surgical complications were noted, with special attention to sinusitis and its symptoms (odor, secretion) and signs (thickening and filling of the sphenoidal cavity), episodes of bleeding or liquorrhea, vision disorders, and meningitis. Tumor volume doubling time (TVDT) was calculated according to Tanaka et al (23). Complete immunohistological examination of the tumorous specimen was performed with quantification of Ki-67 in the tumorous hot spots.

### Statistical analysis

The normality of distribution was tested with the Anderson-Darling test. The data are expressed as the mean ± standard deviations or medians with minimums and maximums. The significance of differences between the groups was assessed with either the *t* test or Wilcoxon test. For pair to pair subgroup testing (consistency/size/radicality), the Pearson and Spearman correlation test and Fisher exact test were used. The level of statistical significance was set at *P* < 0.05. The analysis was conducted with R (R Development Core Team, 2018).

## Results

**Figure 1:**
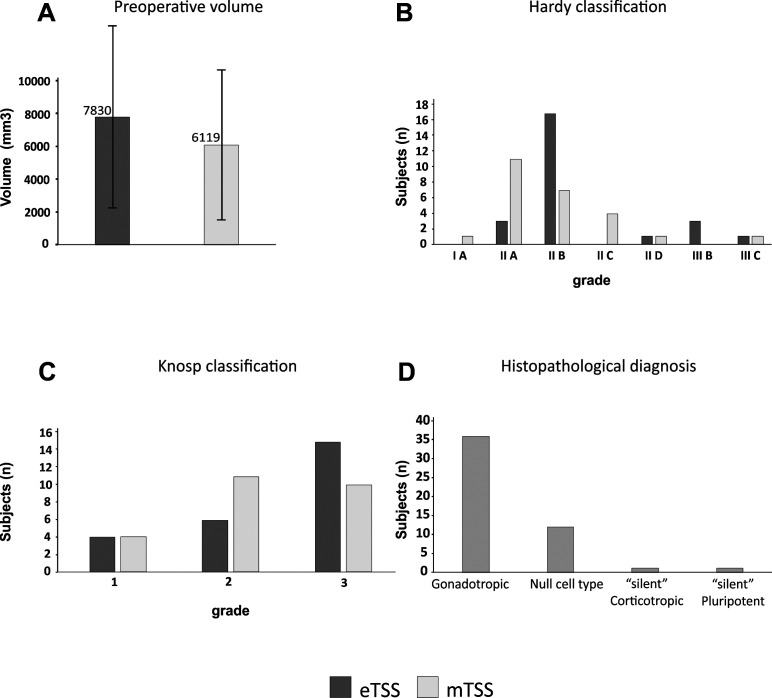
Characterization of non-functioning pituitary adenomas (NFPAs) indicated for surgery. Despite slightly higher initial volume of NFPAs in the endoscopic transsphenoidal surgery (eTSS) group, no significant difference was observed (**A**). The types of NFPAs operated in both groups according to Hardy’s classification (**B**) and Knosp´s classification (**C**). Histopathological diagnosis showed that the majority were gonadotropic adenomas (**D**). For statistical details, please see Table 3.

Patients from the two groups did not differ in age or sex. The eTSS group had significantly shorter postoperative follow-up because the method was introduced only in the last three years of the study (Table 1). The initial volume of NFPAs was slightly higher in the eTSS group, but the difference was not significant (Figure 1A). The most frequent NFPA types in the mTSS group were Hardy II-A and Knosp 2, while the most frequent types in the eTSS group were Hardy II-B and Knosp 3 grades (Figure 1B, Figure 1C). These results indicate that the eTSS group had more parasellar and suprasellar tumor propagation, which is more challenging for the surgeon. According to the histopathological diagnosis, the most common NFPA type was gonadotropic adenoma (Figure 1D).

**Table 1:** Demographic data of patients with non-functioning pituitary adenomas who underwent endoscopic (eTSS) or microsurgical transsphenoidal surgery (mTSS)

	eTSS	mTSS	p
**Sex (M:W)**	16:9	16:9	1*
**Age (years)**	58.7	59.6	0.8^†^
**Follow-up (months)**	23.6	39	<0.001^‡^

*Fisher Exact Test.

†t test.

‡Wilcoxon test.

**Table 3:** Patients’ characteristics and surgical parameters for patients undergoing endoscopic (eTSS) or microsurgical transsphenoidal surgery (mTSS)*^†^

	p	d	N	25q	Median	75q	Mean	SD	Min	Max
**Age (years)**										
eTSS	0.7966	0.07	25	52	60	65	58.68	11.61	36	78
mTSS			25	51	63	66	59.56	12.39	26	78
**Length of the follow-up (months)**										
eTSS	<0.001	1.14	25	15	20	28	23.6	11.93	12	57
mTSS			25	27	39	46	37.16	11.75	18	55
**Preoperative volume (mm^3^)**										
eTSS	0.2799	0.31	25	3910	6100	8980	7830.6	5597.32	1810	23310
mTSS			25	3005	3997	8580	6119.2	4640.92	1170	20700
**Postoperative volume (mm^3^)**										
eTSS	0.7855	0.08	25	0	0	560	302.4	477.76	0	2020
mTSS			25	0	0	210	445.44	1225.3	0	5890
**Volume reduction - overall (%)**										
eTSS	0.9499	0.02	25	93	100	100	95.8	6.16	78	100
mTSS			25	96	100	100	92.72	15.42	38	100
**Volume reduction – group small (%)**										
eTSS	0.9012	0.04	20	94.5	100	100	96.25	5.95	78	100
mTSS			19	96	100	100	92.63	17.04	38	100
**Volume reduction – group large (%)**										
eTSS	1	0	5	93	95	100	94	7.38	82	100
mTSS			6	86.75	98.5	99.75	93	9.84	78	100
**Surgical time (min)**										
eTSS	<0.001	1.87	25	115	120	135	126.8	20.66	95	170
mTSS			25	60	80	85	75.4	21.31	45	125
**Blood loss (mL)**										
eTSS	0.0273	0.64	25	100	200	300	256	195.43	50	900
mTSS			25	100	100	200	156	102.39	50	400
**TVDT/Age**										
TVDT (days)	0.6773	0.09	25	2000.9	4334.25	11048.6	7224	7341.62	502.46	26897.4
age (years)			25	56.25	64	66.5	61.32	11.23	37	78
**TVDT/Ki-67 (days)**										
Ki-67: 0-1	0.3472		10	2520.7	8514.97	12902.1	8609.4	7772.31	535.83	23941.4
Ki-67: 1-2	0.4508		9	1436.5	3433.09	6809.47	4465.9	3961.54	502.46	11504.4
Ki-67: 2-3	0.5628		6	2107	4234.72	10513	9143.5	10514.4	1965.5	26897.4
**Endocrine functions**										
overall		Fisher exact test for count data: *P* = 0.667								
without initial deficit		Fisher exact test for count data: *P* = 0.151								
with initial deficit		Fisher exact test for count data: *P* = 0.063								

*TVDT – tumor volume doubling time; SD – standard deviation.

†Consistency-radicality: Fisher exact test for count data: *P* = 0.449.

**Figure 2:**
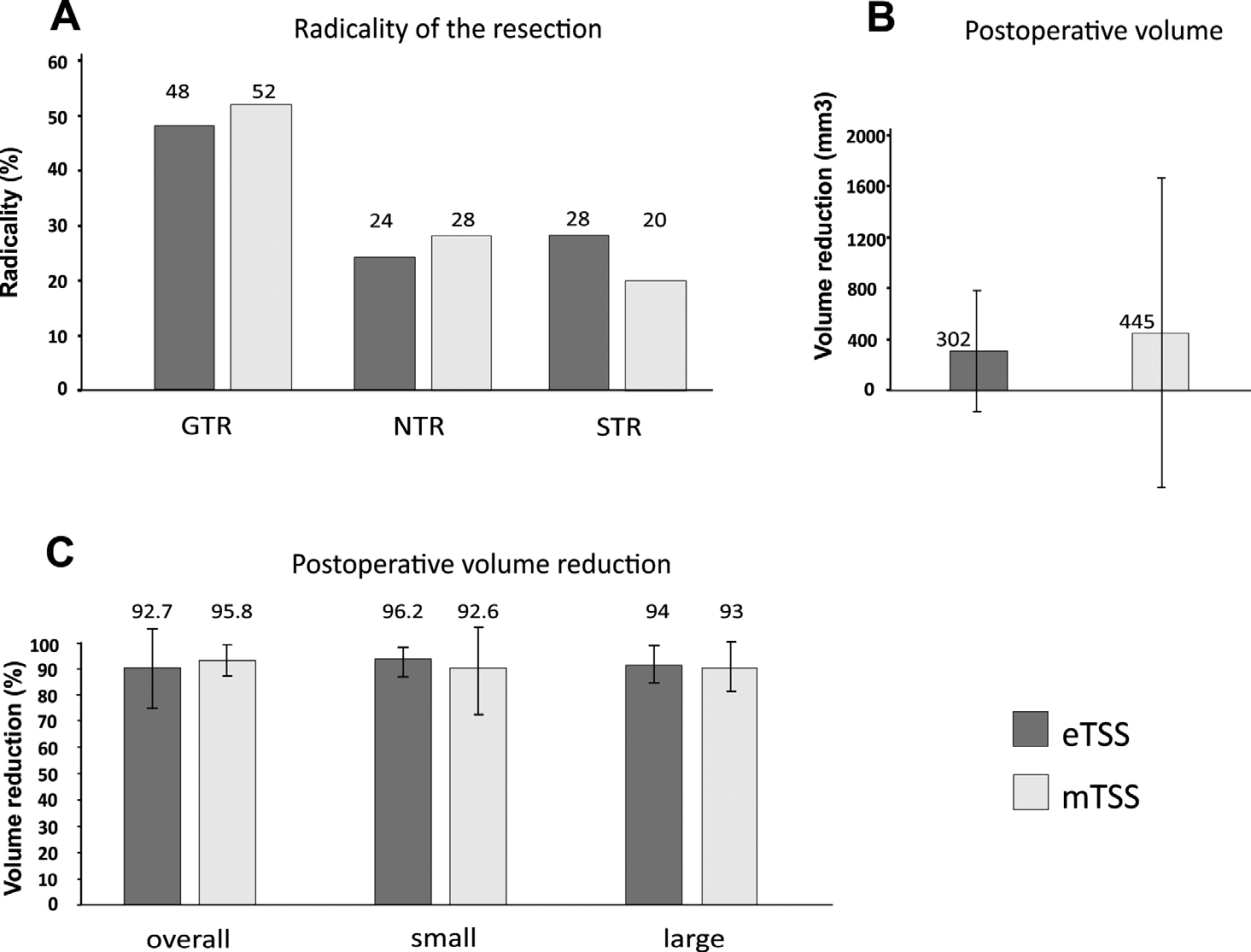
The extent of resection (no residual mass – gross total resection [GTR]; ≤5% of residual mass – near-total resection [NTR]; and ≥5% of residual mass – sub-total resection[STR]) (**A**), residual postoperative volume (**B**), and volume reduction in subgroup analysis (**C**) showed no significant difference between the endoscopic (eTSS) or microsurgical (mTSS) transsphenoidal surgery group. For statistical details, please see Table 3.

The extent of resection was evaluated on the postoperative MRI scans three months after surgery. The groups did not significantly differ in GTR, NTR, or STR resection (Figure 2A). “Satisfactory resection,” defined in most research papers as GTR + NTR, was achieved in 18/25 (72%) patients in the eTSS group and 20/25 (80%) patients in the mTSS group, with no significant difference. No significant differences were also observed in overall volume reduction (Figure 2B and Figure 2C). To determine if the extent of the resection depended on the size of tumorous expansion, we performed a pair-to-pair analysis of “smaller NFPAs” (Hardy IA-IIB, Knosp 0-1) and “larger NFPAs” (Hardy IIC-IIIC, Knosp 2-3) but observed no significant differences between the groups (Figure 2C).

**Figure 3:**
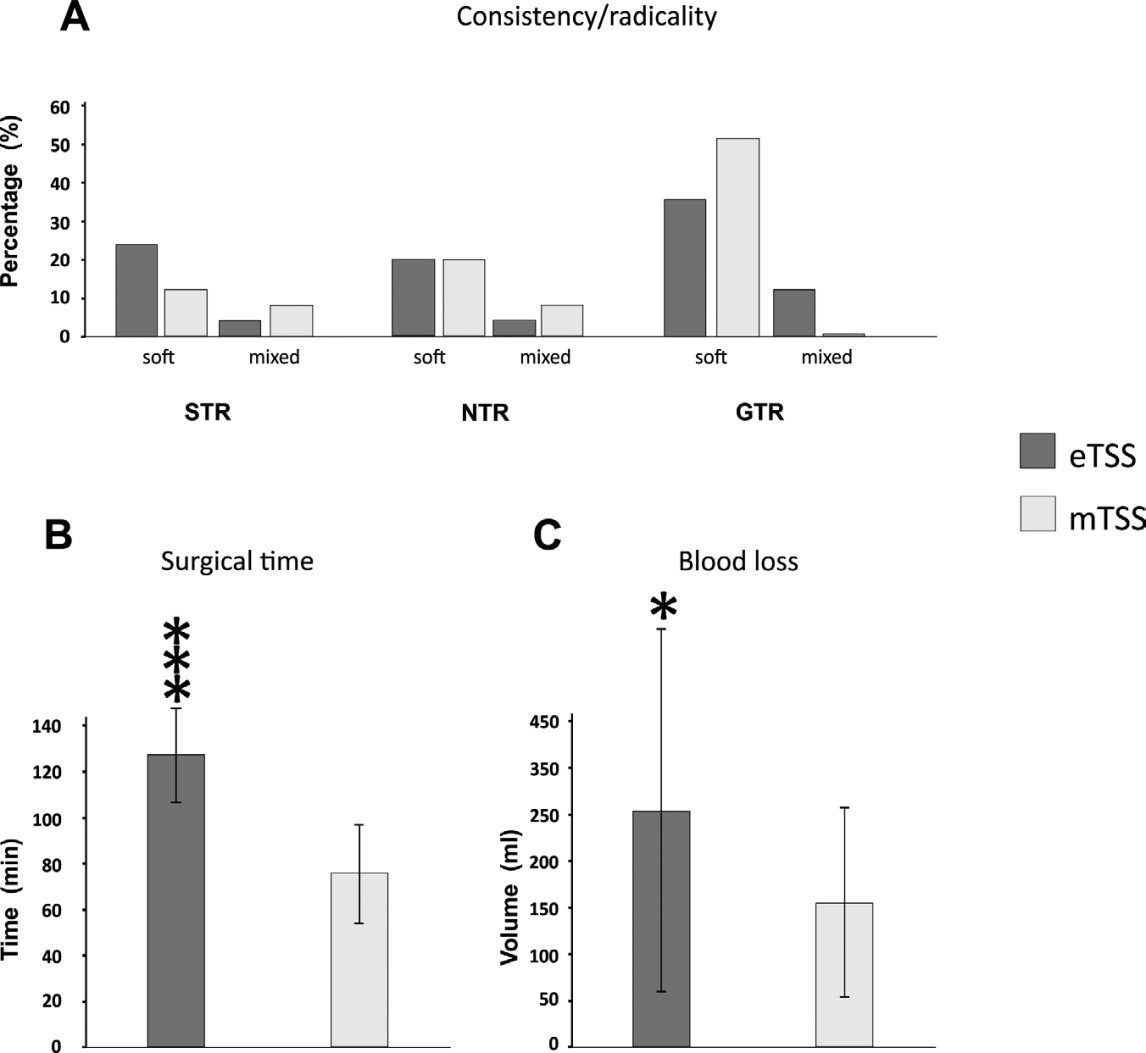
Tumor stiffness can greatly affect the resection feasibility. In both surgical groups we achieved similar rate of resection in accordance with the type of tumor (**A**). The endoscopic transsphenoidal surgery (eTSS) group had significantly longer duration of surgery (**B**) and perioperatively-reported blood loss (**C**). For statistical details, please see Table 3. GTR – gross total resection, NTR – near-total resection, STR – sub-total resection. * *P* < 0.05, *** *P* < 0.001.

We compared the extent of resection with perioperatively reported tumor consistency. No tumor was graded as stiff. Despite a mild reduction of the total number of GTRs in the group of mixed tumors, no significant differences between eTSS or mTSS groups were observed (Figure 3A).

Data on surgery duration and blood loss were obtained from the anesthesia records. The groups significantly differed in mean time of the surgery: 127 minutes in the eTSS vs 75 minutes in the mTSS group (*P* < 0.001) (Figure 3B). The shorter operating time was associated with significantly lower blood loss in the mTSS group (156 mL) compared with the eTSS group (256 mL) (*P* < 0.027) (Figure 3C).

**Figure 4:**
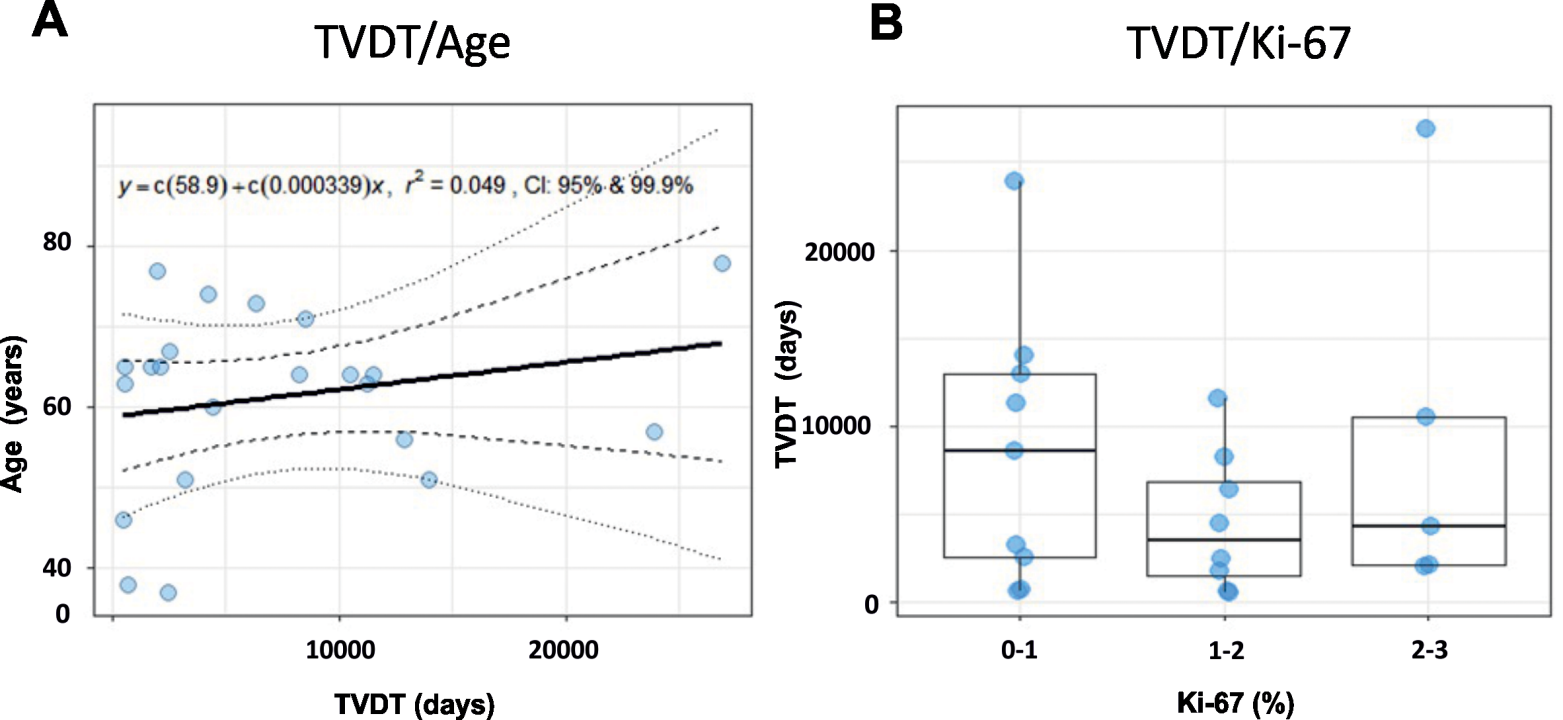
Tumor volume doubling time (TVDT) showed no correlation with age (**A**) or Ki-67 index (**B**). For statistical details, please see Table 3.

TVDT was calculated logarithmically according to the volume of the residual tumorous mass (present in 44% of cases) on postoperative MRI scans. We found no significant correlation between TVDT and patient's age and Ki-67 index (Figure 4A and Figure 4B). Two patients with Ki-67 > 3% showed no signs of relapse during the follow-up. Two more patients (n_eTSS_ = 1, n_mTSS_ = 1) were referred for Leksell gamma knife therapy due to graphical progression of residual disease.

Preoperative visual problems were present in 24/50 (48%) patients, with the majority of them in the eTSS group (17/24 – 70.8%). Most of patients reported subjective improvement in visual acuity three months after surgery, and all patients reported improvement 12 months after surgery. There was no significant difference between the groups.

Chiasmatic syndrome was preoperatively observed in 54% of patients (n_eTSS_ = 19, n_mTSS_ = 8). In the eTSS group, 17 patients reported subjective improvement three months after surgery, 1 patient reported partial improvement 12 months after surgery, and 1 patient, who had pupillary atrophic changes, reported no improvement. In the mTSS group, all patients reported improvement three months after surgery. Despite subjective improvement in the visual field in 26/27 (96.3%) of patients, ophthalmological examination detected discrete blackspots in 4/19 (21%) patients in the eTSS group and 2/8 (25%) patients in the mTSS group, without significant differences between the groups.

**Figure 5:**
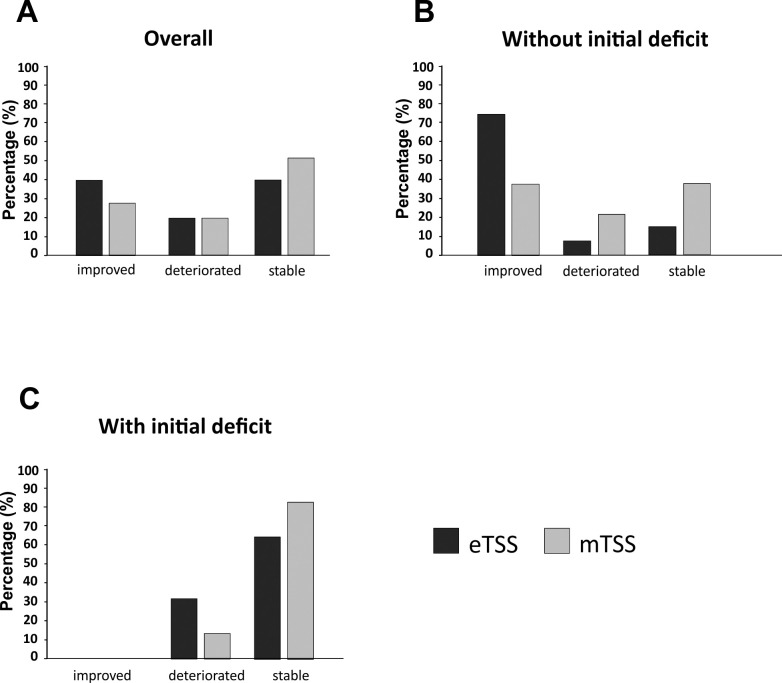
Evaluation of endocrine functions was performed one year after surgery. No significant difference was observed between the groups (**A**). In the subgroup of patients without preoperatively described hormonal deficit, a trend toward improved hormonal profile was observed (**B**). The majority of patients with an initially deteriorated hormonal profile remained hormonally stable (**C**). For statistical details, please see Table 3.

The complete preoperative hormonal profile was compared with the hormonal profile 12 months after surgery to evaluate the endocrine functions. At least one hormonal function improved in 10/25 (40%) of patients in the eTSS and in 7/25 (28%) patients in the mTSS group. No difference in the hormonal profile was identified in 10/25 (40%) patients in the eTSS and 13/25 (52%) of patients in the mTSS group. The remaining 5/25 (20%) of patients in both groups experienced a deterioration in hormonal profile (Figure 5A). We compared the subgroups with or without initial hormonal deficit. In patients without initial deficit we observed a trend toward postoperative improvement of endocrine functions (*P* = 0.15) (Figure 5B). In patients with a deficit, no improvement was observed (Figure 5C). No significant difference between the groups was found.

Seven patients developed postoperative diabetes insipidus (DI). One patient in the eTSS group developed DI 6 months after surgery. Interestingly, no associated image was found on MRI. Another patient developed syndrome of inappropriate antidiuretic hormone release one month after surgery, requiring sodium supplementation. No significant difference in the number of patients with DI or SIADH was found between the groups, even in the subgroup analysis.

In both groups we identified four major complications in the postoperative period (8% rate). One patient from the eTSS group experienced epistaxis, which was resolved during the hospitalization. Three patients developed sphenoidal sinusitis treated by local and systemic antibiotic administration. Two of them, one from each group, were successfully treated during 3-12 months after surgery. One additional patient from the mTSS group was successfully treated within 14 months after surgery. Other minor complications were equally distributed in both groups, with no significant difference (Table 2). Liquorrhea, which was present during the operation in 15 patients (n_eTSS_ = 7, n_mTSS_ = 8), was never detected in the postoperative period.

**Table 2:** The number of minor complications in patients with non-functioning pituitary adenomas who underwent endoscopic (eTSS) or microsurgical transsphenoidal surgery (mTSS)

	_eTSS_	_mTSS_
Nose odor	2	1
Serous secretion	4	4
Mucosa thickening (MRI)	6	13
Serous filling of the sphenoid cavity (MRI)	6	3

## Discussion

The study results showed that eTSS and mTSS had comparable extent of resection, overall clinical or hormonal effect, and complication rate. However, minor differences linked to specific conditions used in our study were found in surgical time and blood loss.

Although a number of studies compared eTSS and mTSS, many of them were criticized for methodological errors, retrospective character, variable definition of radicality of the surgery, or questionable interpretation of visual and hormonal improvement. Moreover, learning curves in the new operating technique considerably vary. Indeed, it is often neglected that surgical experience is probably the most important factor in predicting a favorable outcome (24-26). Familiarization with an endoscope is a difficult and long process due to two dimensional viewing, an absence of depth perception, barrel effect, and clashing instruments. The learning curve for fully endoscopic TSS is between 17 and 34 cases (27). In the light of the learning curve, our results reflect the the greater experience of the surgeon in mTSS than the relatively new eTSS method. These results are comparable with the similarly designed TRANSSPHER study (18).

Generally, radicality of the surgery often depends on tumor size. In mTSS GTR usually ranges from 50% to 75%, whereas in eTSS it ranges from 55% to 95% (11,28-33). A recently published meta-analysis by Almutairi et al, involving nearly 2700 patients, found a significant difference between the techniques, favoring eTSS with 71% of GTR resections against 60.7% in mTSS (14). On the other hand, other authors have failed to prove higher radicality in eTSS (18,20,34-36). This finding is consistent with our results. Reduction of the tumorous mass and the GTR/NTR/STR ratio were similar in both groups. Satisfactory resection, defined as ≥95% reduction in tumor volume was achieved in 72% of eTSS patients and 80% of mTSS patients, a difference that is not significant. Also, unlike other authors, we did not find any significant correlation in the subgroups consisting of only less/more invasive or small/large tumors (37,38). Nevertheless, there was a mild 15% decrease in surgical radicality in the subgroup of invasive NFPAs vs non-invasive NFPAs. Our study indicates that mTSS can be used to successfully treat even NFPAs with more-invasive parasellar behavior. In this respect, we suggest the use of bayonet mirrors, which are very effective in searching for residual tumorous mass.

Despite a mild trend, we also did not observe that tumor consistency depended on surgical radicality. This is probably due to the absence of stiff tumors and the preponderance of mixed tumors, which are relatively “easy” to remove.

A significant difference was observed in surgery duration, which was shorter in the mTSS group. Also, mTSS group had a significantly lower blood loss. Both these findings are in contrast to the results of some previous studies (36,39) but are in agreement with those of the TRANSSPHER study (18). Our findings may be explained by the fact that the larger surgical corridor required in the endoscopic technique, involving time-consuming fenestration of the sphenoid cavity, is performed using the binostral approach. Mastering this technique will probably shorten the surgical time in the eTSS group, as well as the blood loss volume.

Unlike our previous study, this study did not demonstrate any correlation between the growth rate of the residual mass and age or Ki-67 antigen expression (40). However, we believe that previously published growth-rate correlations with Ki-67 have prognostic significance (41-47). It is still a matter of debate whether the limit of 3% of Ki-67 expression proposed by the European Society of Endocrinology is correctly set (48-50). In our opinion, longitudinal prediction of the growth rate of TVDT is an important tool for tailoring the follow-up for surgically as well as conservatively treated patients.

The literature points to major discrepancies in the classification of visual impairments, a problem leading to questionable results interpretation. The effect of the surgery is closely associated with the duration of visual problems, the trophic status of the optic nerves, and comorbidities such as diabetes or arterial hypertension (51,52). In accordance with other studies, we did not find any significant differences between the methods (34,38,53,54). The similar results obtained for both techniques can be attributed to the visual outcome primarily depending on the timing and decompression of the optic apparatus rather than on the extent of the resection itself. Decreased visual acuity, which was reported pre-operatively in 48% of our patients (60% of them in the eTSS group), was improved within the first three months after surgery in 94% of the patients in the eTSS group and in 71.4% of the patients in the mTSS group. One year after surgery, the improvement was observed in all patients. Similarly, chiasmatic syndrome was improved in 96% of patients. These results are in agreement with those of other authors, who reported improvement in 91%-94.7% of patients (20,31,55). Unlike these authors, we did not register any patient with postsurgical visual impairment. Temporary paresis of cranial nerves (abducent and oculomotor nerves) present in three patients was the result of pituitary apoplexy and spontaneously regressed within three months.

Endocrine functions were improved in at least one hormonal function in 34% of patients, with no significant difference between the groups, but with a mild trend favoring the eTSS method. The endoscopic technique is commonly associated with better hormonal outcomes than mTSS (56-58). In contrast, Nomikos et al, in a large series of 660 patients, reported excellent results for mTSS, with nearly 50% improvement and only 1.4% impairment (59). Other studies reported more realistic results – 25%-35% improvement and 29%-32% impairment (60,61).

In our study, surgical complications were equally distributed in both groups. Rhinological complications (bleeding, inflammation) were noted in fewer than 8%, and minor subjective complaints involving nasal discomfort or temporary secretion in 16% of the patients. A higher incidence of postoperative sinusitis after mTSS often mentioned in other studies was not observed in our study. We also do not agree with the rare opinion that microsurgical retraction increases the rate of ischemia of the sinoatrial complex (62,63). Moreover, most of the complications of mTSS were reported when sublabial approach was used, which has been abandoned and replaced by the more gentle paraseptal approach (32,64-66).

Postoperative liquorrhea occurrence in mTSS ranges from 1%-3%, with a higher incidence in eTSS (10,67). Despite a relatively high incidence of peri-operative liquorrhea (30%), we did not observe any postoperative leakage or meningitis, which implies very good sealing of the communication. We also did not register any postoperative visual deficit or major arterial infarction (68-70). Temporary DI was observed in 14% of patients, whereas no patients experienced permanent DI. Permanent DI is reported in 2%-10% of patients undergoing mTSS and in 0%-2% of patients undergoing eTSS (20). In our study, up to 20% patients needed postoperative introduction or increase in hormonal substitutional therapy, a finding that is in agreement with other studies (71,72).

The results of this study have to be interpreted in the light of some limitations. The main limitation is the retrospective study design, which inevitably leads to selection bias. Furthermore, given that eTSS is a recently introduced method, higher surgical experience rate could be expected with the older mTSS method.

In conclusion, our retrospective comparative analysis of transsphenoidal mTSS and eTSS resections of collected NFPAs showed similar results for both procedures in terms of the extent of resection, safeness of the procedures, and overall clinical effect on visual and hormonal functions. mTSS showed significant benefit in terms of surgical time and blood loss. On the other hand, eTSS mildly improved hormonal functions. Despite the overall acceptable surgical outcomes in both techniques, we expect that results of eTSS will improve with increasing experience, and thus this study generally supports our transition to endoscopic pituitary surgery, especially in the cases of large extra- and parasellar tumor extension.
